# Using Exercise and Nutrition to Alter Fat and Lean Mass in Men with Prostate Cancer Receiving Androgen Deprivation Therapy: A Narrative Review

**DOI:** 10.3390/nu13051664

**Published:** 2021-05-14

**Authors:** Rebekah L. Wilson, Dennis R. Taaffe, Robert U. Newton, Nicolas H. Hart, Philippa Lyons-Wall, Daniel A. Galvão

**Affiliations:** 1 Division of Population Sciences, Department of Medical Oncology, Dana-Farber Cancer Institute, Boston, MA 02215, USA; 2 Exercise Medicine Research Institute, Edith Cowan University, Perth 6027, WA, Australia; d.taaffe@ecu.edu.au (D.R.T.); r.newton@ecu.edu.au (R.U.N.); nicolas.hart@qut.edu.au (N.H.H.); d.galvao@ecu.edu.au (D.A.G.); 3 School of Medical and Health Sciences, Edith Cowan University, Perth 6027, WA, Australia; p.lyons-wall@ecu.edu.au; 4 School of Human Movement and Nutrition Sciences, University of Queensland, Brisbane 4072, QLD, Australia; 5 Institute for Health Research, University of Notre Dame Australia, Perth 6160, WA, Australia; 6 Cancer and Palliative Care Outcomes Centre, Queensland University of Technology, Brisbane 4000, QLD, Australia

**Keywords:** androgen deprivation therapy, prostate cancer, exercise, nutrition, fat mass, lean mass

## Abstract

Fat mass (FM) gain and lean mass (LM) loss are common side effects for patients with prostate cancer receiving androgen deprivation therapy (ADT). Excess FM has been associated with an increased risk of developing obesity-related comorbidities, exacerbating prostate cancer progression, and all-cause and cancer-specific mortality. LM is the predominant contributor to resting metabolic rate, with any loss impacting long-term weight management as well as physical function. Therefore, reducing FM and preserving LM may improve patient-reported outcomes, risk of disease progression, and ameliorate comorbidity development. In ADT-treated patients, exercise and nutrition programs can lead to improvements in quality of life and physical function; however, effects on body composition have been variable. The aim of this review was to provide a descriptive overview and critical appraisal of exercise and nutrition-based interventions in prostate cancer patients on ADT and their effect on FM and LM. Our findings are that FM gain and LM loss are side effects of ADT that could be reduced, prevented, or even reversed with the implementation of a combined exercise and nutrition program. However, the most effective combination of specific exercise and nutrition prescriptions are yet to be determined, and thus should be a focus for future studies.

## 1. Introduction

Androgen deprivation therapy (ADT) is a mainstay treatment for prostate cancer (PCa), where more than half of patients will receive ADT at some point during their cancer journey [1]. ADT is a pharmaceutical or surgical strategy that deprives the body of androgens, thereby slowing cancer growth [2]. This may be achieved by either reducing testosterone concentrations to castrate levels defined as <50 ng/dL (<1.7 nmol/L) using luteinizing hormone-releasing hormone agonists, antagonists or an orchiectomy procedure, or by blocking the androgen receptors to eliminate testosterone binding using anti-androgens [2]. Given that testosterone plays roles in the activation of lipolysis and hypertrophy of lean mass (LM) [3,4], substantial body composition changes, as well as loss of muscle strength and physical function, can occur [5,6]. Within the first 9 months of treatment initiation, patients have been reported to experience a 13.8% increase in fat mass (FM) and a 2.4% decrease in LM [5]. This change in body composition places patients with PCa at increased risk of obesity-related comorbidities, treatment-related side effects, development of a more aggressive cancer, and PCa-specific mortality [7,8,9,10].

**Figure 1:**
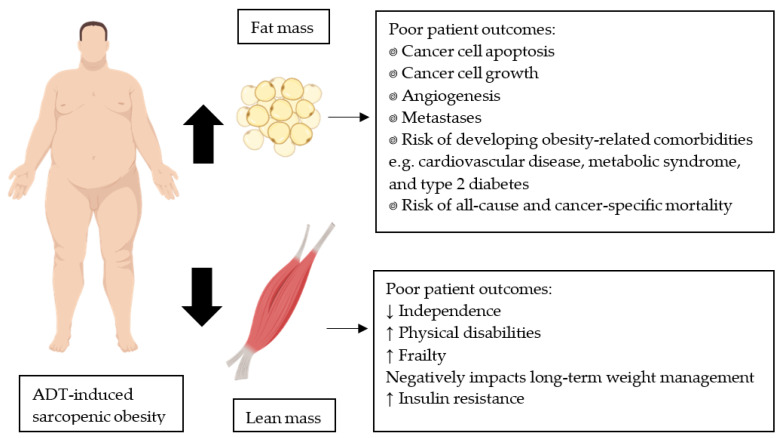
Prostate cancer patients receiving ADT can develop sarcopenic obesity due to a treatment-induced increase in fat mass and decrease in lean mass. These respective body composition changes can lead to poor patient outcomes. Images created with BioRender.com (accessed on 20 November 2020).

Excess FM upregulates pro-inflammatory cytokines, leading to a state of low-grade chronic inflammation, which is associated with decreased cancer cell apoptosis, increased cancer cell growth, angiogenesis, and metastases, and increased risk of developing cardiovascular disease and type 2 diabetes (Figure 1) [7,11,12]. Post-diagnosis obese prostate cancer patients with non-metastatic disease are more likely to experience cardiovascular disease-related mortality than non-obese patients (hazard ratio of 1.24) [13]. In addition, PCa patients on ADT with greater FM may experience higher fatigue, lower vitality, and higher blood triglyceride concentrations [14,15]. A loss of LM also contributes to poorer patient outcomes [14]. The development of sarcopenic obesity, a progressive loss of LM and gain in FM, has been associated with multiple physical disabilities (Figure 1) [16,17]. Lean mass is also the predominant contributor to resting metabolic rate. Therefore, preserving or increasing LM is important for long-term weight loss maintenance [18]. Promoting LM gain can also increase glucose storage, facilitate glucose clearance from circulation, and reduce the amount of insulin required to maintain normal glucose tolerance [19], which is important as insulin resistance may exacerbate cancer progression [20]. Owing to the association between FM gain or LM loss and worse patient outcomes, strategies to prevent or reverse this process are important to include as adjuvant therapies while on ADT, particularly for those who are obese [9].

Exercise and nutrition interventions are effective strategies to reduce FM and increase LM in non-cancer populations [21]. Researchers conducting clinical studies in the PCa population have reported that exercise interventions result in improved quality of life and reduced ADT-related side effects such as cancer-related fatigue and poorer physical function [22]. Nutrition interventions have been demonstrated to induce weight loss, improve bone health, and in some instances slow PCa progression, although evidence is limited [23,24,25]. Despite these beneficial outcomes, the variety of intervention designs, aims, cohorts, and outcomes, presents variable evidence as to whether exercise and nutrition interventions have a desirable effect on FM and LM for patients undergoing ADT. When examining body composition in ADT-treated patients, exercise has been the preferential intervention utilised. As such, there is a lack of clarity concerning the feasibility and efficacy of combined exercise and nutrition programs and the effect on FM loss, while simultaneously seeking to preserve or enhance LM. Therefore, this review is a descriptive overview and critical appraisal of exercise and nutrition-based interventions in ADT-treated PCa patients and the effect on FM and LM, and to propose possible avenues for further research.

MEDLINE and Scopus databases were searched with published studies included until November 2020. Search terms included various combinations of: prostate cancer; androgen deprivation therapy; exercise; nutrition; body composition; fat mass; lean mass. Secondary searches involved reference lists of eligible articles as well as systematic reviews and meta-analyses assessing interventions given to patients on ADT. The key criterion was to identify studies that included PCa patients receiving ADT at time of intervention, utilising an exercise, nutrition or combined intervention, while including a measure of FM and/or LM.

## 2. Using Exercise to Decrease Fat Mass and Preserve or Gain Lean Mass

### 2.1. Aerobic Exercise

Aerobic exercise is an ideal intervention for FM loss as it is familiar to non-exercisers, e.g., walking, easy to implement at home with little to no equipment, promotes higher utilisation of lipids, and includes modes allowing reduced impact on joints, e.g., swimming [26,27]. The aerobic exercise guidelines for prostate cancer patients recommended within clinical practice suggest an accumulation of 150 min/week of moderate-to-vigorous intensity or 300 min/week if weight loss is intended (Table 1) [28]. In this section, we evaluate six studies examining aerobic-based interventions and the effect on FM and LM.

Hvid et al. [29] compared healthy aged-matched controls with normal testosterone concentrations (10–28 nmol/L), and ADT-treated PCa patients with castrate levels of testosterone (<1.7 nmol/L) completing the same 12 week aerobic-based cycling intervention utilising high-intensity interval training (Table 2). Both groups significantly lost whole-body, trunk, visceral, and subcutaneous FM, while preserving LM, with no between-group differences. The castrate levels of testosterone in ADT-treated patients, therefore, does not appear to inhibit FM loss via high-intensity aerobic exercise. However, the healthy controls exhibited a superior loss of intermuscular FM (−8.5% vs. 0%). The presence of substantial intermuscular FM could interfere with muscle fibre quality and contribute to insulin resistance, reduction in muscle strength, and increased fatigue [27,30,31], although there was no between-group difference for insulin sensitivity; muscle strength and fatigue were not measured. However, this study contained a small sample size and did not include a PCa control group. Therefore, it is unclear whether the intervention prevented further ADT-induced increases in intermuscular FM and if this in turn affects muscle fibre quality. Furthermore, the groups had baseline cardiorespiratory fitness levels of 27.2 mL/kg/min and 25.2 mL/kg/min, respectively, and prostate cancer patients staged T1 a/b to T3 a/b. Therefore, the use of high-intensity aerobic-based exercise is uncertain for patients with poor cardiorespiratory fitness or more advanced disease.

Uth et al. [35] utilised an unstructured form of interval-based aerobic training, in the form of football (soccer) game play and skill development (Table 2). Unlike Hvid et al. [29], they recruited patients with bone metastases (19.3%), but similarly assessed an apparently healthy prostate cancer cohort with only 5.3% of patients self-reporting a sedentary lifestyle, with baseline cardiorespiratory fitness of 27.2 and 26.4 mL/kg/min, and mean body mass index of 26.7 and 27.6 kg/m^2^, respectively. They reported a mean 0.5 kg significant increase in LM and a mean 0.6 kg loss of FM that approached within-group significance. With the improvement in LM and a trend for an effect on FM, sport-orientated activities may be an effective alternative to clinic-based interventions in ameliorating treatment-related body composition changes. Several adverse events were reported in the football group including fracture, tendon tear, and sprain. While no injury was related to bone metastases and most participants recovered and continued with the study, there is uncertainty whether such an intervention would be feasible for high-risk patients, e.g., obese patients with multiple comorbidities. Injury risk is higher within a team sport environment, compared to individual sport or exercise, due to the unpredictable nature of opponents, teammates, and ball. The authors suggested a lead-in period may be required to improve strength, balance, and ball handling to reduce injury risks [35].

In contrast to the previous studies using interval training [29,35], Newton et al. [36] and Alberga et al. [32] utilised clinic-based continuous aerobic exercise (Table 2). Examining a cohort that excluded patients with bone metastases, Newton et al. [36] used a three-arm study design over 12 months comparing impact and resistance exercise, aerobic and resistance exercise, and delayed aerobic exercise after 6 months of usual care. When compared to the aerobic-only exercise group during the 6–12 month period, no differences in FM or LM were noted between groups. Alberga et al. [32] also utilised a three-arm study design comparing aerobic exercise, resistance exercise, and usual care across a 24-week period, in ADT and non-ADT groups, although the two treatment types were not compared. The ADT aerobic group exhibited an undesirable significant increase in body fat percentage (BF%) and 2 kg reduction in LM, although not statistically different to the other ADT groups. The researchers did not report FM, so it is unclear whether a change in FM, in addition to the LM loss, contributed to the modification in BF%. The decline in LM is substantial and concerning, suggesting the prescribed aerobic exercise was insufficient to prevent ADT-related declines in LM, in contrast to a non-significant 0.5 kg loss in LM in the non-ADT aerobic group.

The previously described studies were supervised interventions [29,32,35,36]. However, ongoing supervision is not always viable. Santa Mina et al. [33,34] compared home-based aerobic and resistance exercise over 6 months examining patients with non-metastatic disease. Santa Mina et al. [34] used a smaller non-randomised group of the same cohort to report on blood biomarkers (Table 2). There were significant within-group declines in chest skinfold thickness and BF% at 3 months, but not 6 months [33] and weight change was positively associated with changes in leptin and the leptin:adiponectin ratio, and negatively associated with IGF-1:IGFBP-3 ratio [34], which are proposed markers associated with PCa progression [45]. Although the use of anthropometric measures suggest weight loss may improve risk of cancer progression, the researchers could not confirm if these changes were subject to alterations in FM or LM. Nonetheless, both studies provide valuable insight into the potential of home-based programs, although there is still uncertainty if those with metastatic disease would benefit from a similar program.

### 2.2. Resistance Exercise

Weight loss can occur through loss of both fat and muscle tissue [46]; however, substantial loss of LM may exacerbate sarcopenia, reduce physical function, and increase risk of falls [47]. Resistance exercise is commonly prescribed for muscle hypertrophy [48]. Within clinical practice prostate cancer patients are recommended to complete resistance training on a minimum of two days each week (Table 1) [28]. This section is an evaluation of six studies examining resistance exercise and the effect on FM and LM.

Galvão et al. [37] and Hanson et al. [38] conducted single-group studies and both excluded patients with metastatic disease (Table 2). Galvão et al. [37] prescribed a traditional periodised resistance training program over 20 weeks and found no change in FM or LM except for a significant increase in quadriceps thickness. In contrast, Hanson et al. [38] utilised drop sets and repetitions to failure over a 12-week program. The exercise set began at five repetition maximum and once volitional fatigue was reached the resistance was reduced until 15 repetitions were achieved. A significant decrease in BF% and increase in LM were reported. The differing results may be explained by the period between the two studies and cohort examined. At the time of the Galvão et al. [37] study, the use of resistance training for PCa patients was somewhat revolutionary and a conservative exercise prescription was implemented with only 10 patients recruited. The Hanson et al. [38] study was completed over a decade later in a cohort of 17 patients of African American ethnicity with higher intensity and sophistication of resistance training design. While these studies demonstrate the feasibility of resistance training in promoting changes to LM, both studies utilised small or non-diverse cohorts, so the generalisability of these results is unclear.

Nilsen et al. [39] examined a 16-week clinic-based high-load periodised resistance training program in which the intervention group significantly improved appendicular skeletal muscle (ASM). However, no changes were found for whole-body LM or FM or for any body composition measure when compared to the usual care controls (Table 2). High-risk patients with medical conditions that could complicate participation were excluded from this study, although cancer stage of included patients was not reported. Nevertheless, three patients withdrew from the intervention group due to pain. Further research is required into the appropriateness of high-load resistance training for high-risk patients and may require a gradual increase in intensity. Furthermore, while the recruitment goal was met in this study, the authors reported to be uncertain whether the effect size selected to calculate sample size was appropriate to detect a change in LM.

Resistance and aerobic exercise are both recommended in the PCa survivorship guidelines [28]. Therefore, it is important to understand how patients respond to each exercise mode. Alberga et al. [32] and Santa Mina et al. [33,34] compared aerobic and resistance exercise (Table 2). Alberga et al. [32] utilised clinic-based periodised resistance training conducted over 24 weeks and reported preservation of BF% and LM, which was significantly different to usual care controls who gained BF% and lost LM. The 2 kg LM loss in the aerobic group although not statistically different to the 0.3 kg loss in the resistance group, is of clinical relevance and highlights the importance of resistance training in preserving LM. Santa Mina et al. [33,34] examined home-based resistance exercise utilising bands, balls, and body weight exercises, and reported no training effect [33,34]. From this work, it appears that resistance training alone is insufficient to induce FM loss. However, it may prevent further ADT-induced body composition changes and specifically alleviate loss of LM.

### 2.3. Multi-Modal Interventions

The inclusion of multiple exercise modes is important when the intention is to alter both FM and LM. In this section, we evaluate seven studies utilising multi-modal interventions and the effect on FM and LM.

Several authors examined similar cohorts without bone metastases and compared combined aerobic and resistance exercise interventions to usual care controls (Table 2). Galvão et al. [40] reported significant between-group differences in whole-body LM and ASM, but no change in FM over 12 weeks. Cormie et al. [41] found significant between-group differences for whole-body and trunk FM, BF%, and ASM over the 12-week intervention. The intervention group demonstrated a significant within-group loss of visceral FM, while the control group significantly lost LM, ASM, and gained whole-body FM and BF%. Wall et al. [43] reported significant between-group differences for whole-body FM and LM, trunk FM, and BF% but conducted a longer intervention of six months. Ndjavera et al. [44] reported no body composition changes over their 12-week intervention. Cormie et al. [41], Wall et al. [43], and Ndjavera et al. [44] reported greater adjusted group mean differences for FM (−1.4, −1.1, and −1.9 kg, respectively) than Galvão et al. [40] (−0.01 kg), which could be explained by the larger volume of aerobic exercise prescribed in these studies.

Galvão et al. [15] was a secondary analysis of the previously described Galvão et al. [40] study and they compared different durations of ADT: chronic ≥ 6 months, and acute < 6 months, completing the same intervention (Table 2). The authors reported a significant between-group difference in FM with those on chronic ADT experiencing a 0.4 kg loss compared to a 0.6 kg gain in the acute ADT group over 12 weeks. Furthermore, triglyceride concentrations were significantly different between groups, which was associated with the observed changes in FM. Despite these significant findings it resulted in an uneven distribution between acute (*n* = 16) and chronic (*n* = 34) ADT-treated patients due to the use of a delayed exercise control group. The smaller number in the acute group may have limited the ability to observe differences between groups. Regardless, it is important to note that body composition declines are greater during the initial 3–6 months of ADT commencement and appear more difficult to ameliorate with exercise therapy.

Aerobic and resistance-based exercise are the most commonly prescribed modes; however, both Newton et al. [36] and Winters-Stone et al. [42] examined the combined effect of impact training, e.g., bounding movements, and resistance training (Table 2). Newton et al. [36] reported that the combined impact/resistance group significantly improved ASM compared to the usual care controls at 6 months. However, no effect on ASM was noted after the same resistance training was undertaken by the aerobic/resistance group. The authors described a potential interference effect when combining aerobic and resistance training within the same session, which may have compromised muscle hypertrophy [49]. Winters-Stone et al. [42] reported that FM was significantly decreased in the impact/resistance group compared to a flexibility control group who continued to gain FM. Additionally, in line with the Santa Mina et al. [34] findings, Winters-Stone et al. [42] reported that the changes in FM mediated differences in insulin, suggesting FM loss induced an insulin-lowering effect.

## 3. Using Nutrition to Decrease Fat Mass and Preserve or Gain Lean Mass

### 3.1. Healthy Eating Guidelines and/or Energy Deficit

**Figure 2:**
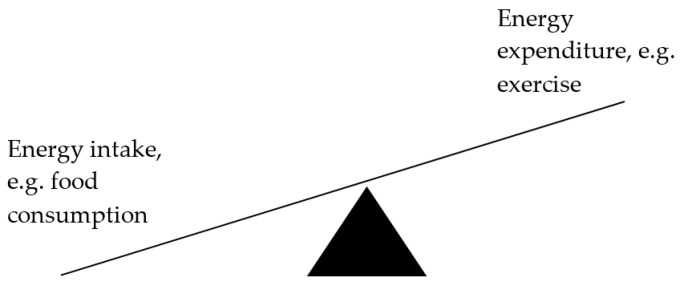
Weight loss occurs when energy expenditure is greater than energy intake.

Healthy eating guidelines are recommended portions of each food group to be consumed daily [50]. Weight loss in its simplest form is achieved through greater energy expenditure over intake creating a daily energy deficit (Figure 2) [51]. Clinical practice guidelines recommend prostate cancer patients to consume a healthy balanced diet high in fruit and vegetables, low in saturated fat, and consume adequate amounts of vitamin D (>600 IU) and calcium (<1200 mg/d), with an energy deficit if weight loss is required (Table 1) [28]. In this section, we review six studies in which healthy eating guidelines and/or an energy deficit were implemented and the effect on FM and LM evaluated.

Gilbert et al. [52] and Focht et al. [53] prescribed combined aerobic and resistance-based exercise and conducted small group healthy eating seminars over a 12-week period (Table 3). Gilbert et al. [52] reported a significant difference in LM but no change in FM compared to usual care controls. However, the intervention group reduced their mean FM from 34.5 to 31.6 kg compared to 30.4 to 29.0 kg in the control group. Although the 2.9 kg FM loss for the intervention group is potentially clinically meaningful, no within-group changes were reported. Focht et al. [53] additionally included group-mediated behaviour modification seminars based on social cognitive theory. Compared to usual care controls, the intervention group significantly lost FM and BF%, with no change in LM. Although the exercise and nutrition sessions were well adhered to, only a small subset of the patients provided 3 day weighed food records and, therefore, overall nutritional intake and compliance to nutrition advice were not confirmed. Further, 80% of patients in the intervention group were overweight or obese and prescribed an energy deficit diet. Therefore, the contribution of healthy eating guidelines versus an energy deficit diet to promote FM and LM changes is unclear.

O’Neill et al. [54] prescribed a 6-month home-based walking program, with a dietary booklet encouraging healthy eating habits to patients of all cancer stages (T1–4), although metastatic status was not reported (Table 3). The authors reported a significant reduction in FM and BF%, with no change in LM when compared to usual care controls. While they showed that a home-based intervention can reduce FM, body composition was measured using the less precise technique of skinfold measurement. Similarly, to Focht et al. [53], O’Neill et al. [54] encouraged an energy deficit diet only for patients who were overweight or obese.

Freedland et al. [55] and Wilson et al. [57] targeted overweight or obese patients who did not have symptomatic or bone metastases, respectively (Table 3). Freedland et al. [55] prescribed home-based walking and a low carbohydrate diet over 6 months. Compared to an 11% increase in FM for the usual care controls, the intervention group significantly lost 16.2%. This substantial loss in FM has not been previously achieved in PCa patients on ADT. However, the intervention group also had a significant decline in LM compared to controls. A loss in LM is not uncommon while undergoing weight loss [46], with similar patterns also noted by Baguley et al. [56] in their 12-week nutrition-only intervention (Table 3). Wilson et al. [57] also demonstrated a significant reduction in FM but in contrast, achieved LM preservation. Wilson et al. [57] included supervised resistance training and protein supplementation, which are both considered important for LM preservation [59]. While the intervention designs are different, these studies provide preliminary evidence on the potential for effective FM and LM management for obese ADT-treated PCa patients through diet and exercise, which includes resistance training.

### 3.2. Protein Intake

The optimisation of protein intake is often incorporated into weight loss nutrition plans to assist the body to mobilise fat and preserve muscle tissue by supporting the upregulation of muscle protein synthesis [59]. Next, we describe a study examining protein supplementation and resistance exercise.

Dawson et al. [58] examined four groups of patients with PCa, including those with metastatic disease (54.3%), over a 12-week period: exercise-only, exercise and protein supplement, protein supplement-only, and usual care control (Table 3). No additional effect was found for protein supplementation and as the study was not powered to detect changes using a four-armed design, results were reported for exercise versus non-exercise groups. In the exercise groups there was a significant increase in LM, ASM, and fat-free mass, a significant reduction in BF%, with no changes in FM. The lack of a synergistic effect of protein supplementation could be attributed to the low adherence of the protein-only group who consumed 1.0 g/kg/day compared to 1.1–1.4 g/kg/dayin the other three groups. Further, the protein supplements were given as 2 × 25 g daily doses. This may not have been sufficient to stimulate muscle protein synthesis as each dose was equivalent to ~0.3 g protein/kg body weight/day, compared to the ~0.4 g protein/kg body weight/daywhich has been shown to be effective in increasing muscle protein synthesis when combined with an acute bout of resistance exercise in ADT-treated PCa patients [59].

## 4. Discussion

The field of exercise oncology has rapidly developed over the last two decades and we have presented 22 exercise and nutrition interventions conducted in ADT-treated PCa patients between 2006 and 2020. Despite this growth in awareness of the benefits that can be derived from undertaking these practices, most of the studies report only modest changes in FM and LM. In this discussion, we summarise the key conclusions from these studies and propose future research directions to progress the field.

The American Cancer Society weight loss guidelines for PCa patients are no different to that of the general population (Table 1) [28]. Notably, Wilson et al. [57] was the only study to incorporate these guidelines, which are recommended in clinical practice but have not been verified in the ADT-treated population. Although these guidelines have the potential to provide successful body composition changes, the metabolic changes induced by ADT likely require different strategies to induce change compared to the non-ADT population, as alluded to by the results of Alberga et al. [32], although the ADT and non-ADT cohorts were not compared. In this regard, we provide an important initial platform to help identify how these guidelines may be tailored to suit hypogonadal men. Potential questions that would lead to further understanding of how to tailor these weight loss guidelines for ADT-treated patients to maximise FM and LM changes are presented in Table 4.

With body composition changes occurring early in the treatment process [60], it would be preferable to implement an exercise and nutrition intervention at initiation of ADT. However, the magnitude of intervention-induced body composition changes could depend on length of time on ADT, as demonstrated by Galvão et al. [15], where those initiating ADT may experience small or no intervention-induced changes compared to those on chronic ADT. Similarly, Hvid et al. [29] highlighted a patient on ADT for <6 months who did not respond to the exercise intervention and gained 2.6 kg of FM accompanied by a loss in LM of 5.0 kg. Ndjavera et al. [44] also reported no training effect on body composition within the first 3 months of ADT. However, each of these studies were exercise only and it has been established that manipulation of nutrition substantially decreases FM more than exercise alone [61]. Therefore, those initiating ADT may only experience substantial FM loss when nutrition is also addressed, as was demonstrated by Freedland et al. [55]. Regardless of the influence of length of time on ADT on body composition changes, exercise and nutrition should still be recommended from therapy onset as there will be additional health benefits and likely prevention of substantial FM and LM changes, as demonstrated by Cormie et al. [41].

Studies utilising a multi-modal intervention compared to a single-exercise mode showed more consistent beneficial responses in both FM and LM. However, the majority of the multi-modal studies were conducted by the same research group [15,36,40,41,43,57] and, therefore, may not represent the wider PCa population. Capitalising on the unique benefits gained from utilising multiple exercise modes can induce concurrent desired adaptations of FM and LM. However, there is uncertainty of best practice regarding exercise prescription to induce concurrent FM loss and LM preservation or gain. While high-intensity [29,35,38] and high-volume [54,55,57] exercise resulted in the greatest changes in FM or LM, they may not initially be suitable for obese patients who have multiple comorbidities without undergoing a lead-in phase to improve baseline fitness. Moreover, the impact of such interventions on patients with metastatic disease is unclear with only two studies actively recruiting patients of this disease stage [35,58]. Further research is required into the benefits of high-intensity or interval-based interventions, such as high-intensity interval training or team/individual sports, for ADT-treated PCa patients. There may also be a minimum-intensity threshold that stimulates lipolysis and muscle protein synthesis, as demonstrated by Alberga et al. [32], where patients undertaking aerobic exercise continued to gain BF% and lose LM. Furthermore, the use of multiple modes within the same session, as noted by Newton et al. [36], may have an interference effect where physiological pathways involved in manipulating body composition are not stimulated compared to when a single-exercise mode is undertaken.

While bone measurements are not reported in the current review, it is important to highlight that in addition to FM gain and LM loss patients receiving ADT may also experience a loss of bone mass placing them at increased risk of osteopenia or osteoporosis [5]. Newton et al. [36] assessed bone health as their primary outcome and reported preliminary efficacy for the inclusion of impact training in a multi-modal intervention to prevent ADT-induced bone loss. Patients at increased risk of bone loss may also benefit from increased calcium and vitamin D intake, which are included as part of the exercise and nutrition guidelines for prostate cancer patients [28].

The number of interventions measuring body composition that encompassed a nutrition component were less common than those investigating exercise. The employment of an energy deficit was effective at reducing FM as shown in both the O’Neill et al. [54] and Freedland et al. [55] studies. However, preventing LM loss when the body enters a catabolic state requires further clarity. Protein optimisation and the inclusion of resistance training may be important components to promote LM preservation or gain when undergoing weight loss as suggested by Wilson et al. [57] and Dawson et al. [58]. However, as protein supplementation is currently understudied in this population, it is not included in the PCa weight loss guidelines and needs further evaluation. Continued research into optimal diet and exercise prescriptions for prostate cancer patients may further improve the benefits of weight loss and the potential impact on a patient’s prognosis with particular interest in diet and exercise modes that influence microbiome activity. Differences in composition of the gut microbiome have been reported in men with prostate cancer compared to men with benign prostatic conditions and could contribute to prostate cancer pathogenesis and progression [62].

As noted by Nilsen et al. [39], the definition of a clinically significant change in FM and LM needs to be established. A 5% loss of body weight, which should be predominantly FM loss [63], has been shown in the non-cancer population to improve blood pressure, cholesterol, and insulin resistance [64]. While this percentage is also used for cancer patients, the significance is unknown. For example, increases in trunk, visceral, and intermuscular FM are associated with increased insulin resistance, a potential mechanism for the observed association between FM and PCa progression [65,66]. Therefore, a loss of FM in these regional areas, independent of whole-body FM loss, may be more beneficial for PCa patients on ADT than a 5% loss in total body mass [29,63]. Further, it is unknown whether a loss in FM will improve a PCa patient’s risk of disease progression, treatment-related side effects, or comorbidity development. Both Santa Mina et al. [34] and Winters-Stone et al. [42] demonstrated that weight or FM loss was associated with improvements in biomarkers related to cancer progression, which has also been demonstrated in non-ADT PCa patients [67]. Moreover, Galvão et al. [15] reported that a decrease in FM was associated with decreased serum triglyceride levels. These studies provide preliminary evidence that FM loss could improve patient outcomes.

## 5. Conclusions

Fat mass gain and LM loss are side effects of ADT that might be prevented or reversed with the implementation of an exercise and nutrition intervention. Patients on ADT, particularly those who are obese, require effective strategies to improve their body composition, which in turn may improve general health and cancer-free survival. The implementation of such strategies will be most successful through the effective communication of a multi-disciplinary team including, but not limited to, oncologists, urologists, dietitians, and exercise physiologists. The inclusion of a multi-modal exercise program is needed to stimulate both lipolysis and muscle protein synthesis to ensure FM loss and LM preservation. While exercise should be tailored to the preferences and fitness level of the patient, when FM loss is the objective, energy expenditure should be maximised, which is best achieved through higher volume and intensity with the inclusion of an energy deficit diet. The optimal macronutrient composition of a diet for PCa patients on ADT is unclear but should ultimately follow healthy eating guidelines and optimise protein intake.

## Figures and Tables

**Table 1:** Current prostate cancer-specific exercise and nutrition guidelines, including weight loss guidelines.

	Current Exercise and Nutrition Guidelines	Current Weight Loss Guidelines
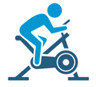 Aerobic training	150 min/week of moderate intensity exercise or 75 min/week of vigorous intensity exercise	300 min/week of moderate intensity exercise or 150 min/week of vigorous intensity exercise
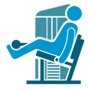 Resistance training	Minimum two strength training sessions/week	
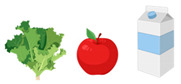 Nutritional intake	Healthy balanced diet with high fruit and vegetables, low saturated fats, and adequate calcium (<1200 mg/d) and vitamin D (>600 IU)	2100–4200 kJ daily energy deficit

Images created with BioRender.com (accessed on 5 April 2021).

**Table 2:** Exercise-only interventions assessing fat and lean mass in prostate cancer patients receiving ADT.

Study	Study Design	Primary Outcome	Intervention	Body Composition Assessment	Groups (*N*)	Outcome Variable	Mean Pre-Intervention Values (kg)	Mean Post-Intervention Values (kg)
Aerobic-based interventions								
Alberga et al. [32]	RCT	Body composition and fitness	24 weeks 3 ×/week Supervised aerobic exercise at 50–75% HRmax or Supervised resistance exercise at 60–70% 1 RM	DXA	Aerobic (*N* = 40)			
					ADT	BF%	31.2%	33.3% *
						Lean mass	65.0	63.0 *
					No ADT	BF%	29.9%	30.5%
						Lean mass	66.2	65.7
					Resistance (*N* = 40)			
					ADT	BF%	32.6%	33.0% §UC
						Lean mass	63.7	63.4 §UC
					No ADT	BF%	29.7%	29.2%
						Lean mass	66.7	67.3
					Usual care (*N* = 41)			
					ADT	BF%	32.0%	35.2% §R *
						Lean mass	64.2	61.1 §R *
					No ADT	BF%	31.2	30.6
						Lean mass	65.0	65.6
Hvid et al. [29]	Prospective cohort	Insulin sensitivity and body composition	12 weeks 3 ×/week 135 min/week Aerobic interval exercise 50–100% VO2max	DXA and MRI	Prostate cancer exercise (*N* = 9)	Fat mass	24.4	23.1 #
						Trunk fat	14.5	13.4 #
						Lean mass	52.3	52.3
						BF%	31.1%	29.8% #
						Visceral ^a^	−8.4% #	
						Subcutaneous ^a^	−4.9% #	
						Intermuscular ^a^	0% § #	
					Non-cancer exercise (*N* = 10)	Fat mass	20.5	19.6 #
						Trunk fat	12.4	11.8 #
						Lean mass	56.3	56.2
						BF%	25.7%	25.0% #
						Visceral ^a^	−5.8% #	
						Subcutaneous ^a^	−2.5% #	
						Intermuscular ^a^	−8.5% #	
Santa Mina et al. [33]	RCT	Quality of life	6 months 3–5 ×/week 90–300 min/week Home-based resistance band/ball/body weight exercise 12–15 RPE Or Home-based aerobic exercise at 60–80% HRmax	Skinfolds	Aerobic (*N* = 22)	Chest skinfold	35.6 mm	33.5 mm *3
						BF%	28.5%	27.3% *3
					Resistance (*N* = 34)	Chest skinfold	35.3 mm	33.7 mm
						BF%	28.0%	27.3%
Santa Mina et al. [34]	RCT	Blood biomarkers	See Santa Mina et al. [33]	Skinfolds	Aerobic (*N* = 13)	BF%	28.4%	26.4%
					Resistance (*N* = 13)	BF%	26.5%	25.3%
Uth et al. [35]	RCT	Lean mass	12 weeks 2 ×/week (1–8 weeks) 3 ×/week (9–12 weeks) 90–180 min/week Supervised football training	DXA	Football (*N*= 29)	Fat mass	27.6	26.3
						Lean mass	53.1	54.0 § *
						BF%	32.6%	31.7%
					Usual care (*N* = 28)	Fat mass	30.0	29.7
						Lean mass	56.7	56.8
						BF%	32.9%	32.9%
Newton et al. [36]	RCT	Bone mineral density	12 months 2 ×/week Supervised impact exercise at ground reaction force of 3–5 × body weight Resistance exercise 6–12 RM, 2–4 sets 2 ×/week Home-based impact exercise Or 6 months 2 ×/week 150 min/week Supervised aerobic at 65–85% HRmax Resistance exercise 6–12 RM, 2–4 sets Home-based aerobic exercise 6 months Home-based aerobic Resistance (body weight/band) exercise Or 6 months waiting period 6 months 2 ×/week 80 min/week Aerobic exercise at 70% HRmax	DXA	Resistance/impact (*N* = 57)	Fat mass	24.0	25.1
						Lean mass	57.9	59.3
						ASM	25.0	25.9 §6DEL
					Aerobic/resistance (*N* = 50)	Fat mass	22.8	23.7
						Lean mass	58.1	58.7
						ASM	25.2	25.6
					Delay/aerobic (*N* = 47)	Fat mass	27.1	28.3
						Lean mass	59.3	60.4
						ASM	25.3	25.9
Resistance-based interventions								
Galvão et al. [37]	Prospective cohort	Muscle function	20 weeks 2 ×/week 120 min/week Supervised resistance exercise 6–12 RM, 2–4 sets	DXA	Resistance (*N* = 10)	Fat mass	25.7	24.9
						Lean mass	52.2	52.0
						BF%	30.7%	30.6%
						Quadriceps thickness	2.15 cm	2.46 cm *
						Hamstring thickness	4.52 cm	1.53 cm
						Biceps thickness	2.69 cm	2.91 cm
						Triceps thickness	1.94 cm	2.33 cm
Alberga et al. [32]	Details in aerobic section							
Santa Mina et al. [33]	Details in aerobic section							
Santa Mina et al. [34]	Details in aerobic section							
Hanson et al. [38]	Prospective cohort	Muscle size and function	12 weeks 3 ×/week 180 min/week Supervised high-intensity resistance exercise 15 repetitions, first 5 at 5 RM	DXA and CT	Resistance (*N* = 17)	Fat mass	31.2	31.1
						Subcutaneous	118 cm^2^	118 cm^2^
						Intermuscular	7.9 cm^2^	7.6 cm^2^
						Lean mass	62.4	64.1 *
						BF%	31.4%	30.7% *
Nilsen et al. [39]	RCT	Lean mass	16 weeks 3 ×/week Supervised resistance exercise 6–10 RM, 1–3 sets	DXA	Resistance (*N* = 28)	Fat mass	26.5	26.4
						Trunk fat	14.7	14.6
						Lean mass	59.8	60.3
						ASM	25.2	25.7 §
						BF%	29.5%	29.3%
					Control (*N* = 30)	Fat mass	26.4	26.7
						Trunk fat	14.6	14.7
						Lean mass	57.9	57.9
						ASM	24.8	24.7
						BF%	30.0%	30.2%
Multi-modal interventions								
Galvão et al. [40]	RCT	Lean mass	12 weeks 2 ×/week Supervised aerobic at 65–80% HRmax Resistance exercise 6–12 RM, 2–4 sets	DXA	Exercise (*N* = 29)	Fat mass	22.5	22.3
						Trunk fat	12.2	11.9
						Lean mass	56.1	56.8 §
						ASM	23.5	24.0 §
						BF%	27.5%	27.2%
					Usual care (*N* = 28)	Fat mass	23.2	23.5
						Trunk fat	12.4	12.2
						Lean mass	57.8	57.8
						ASM	24.6	24.4
						BF%	27.3%	27.5%
Galvão et al. [15]	RCT	Various ADT side effects	See Galvão et al. [40]	DXA	Acute ADT (*N* = 16)	Fat mass	22.7	23.3 § *
						Trunk fat	12.2	12.4
						Lean mass	58.5	59.1
						ASM	24.7	25.2
						BF%	26.8%	27.2% §
					Chronic ADT (*N* = 34) ^b^	Fat mass	23.4	23.0 *
						Trunk fat	12.1	11.8 *
						Lean mass	56.5	57.4 *
						ASM	23.8	24.4 *
						BF%	28.1%	27.4% *
Cormie et al. [41]	RCT	Lean mass	12 weeks 2 ×/week 150 min/week Supervised aerobic at 70–85% HRmax Resistance exercise at 60–85% 1 RM Home-based exercise of choice	DXA	Exercise (*N* = 32)	Fat mass	26.9	26.3 §
						Trunk fat	14.8	14.3 §
						Visceral fat	913 g	874 g *
						Lean mass	56.6	56.0
						ASM	23.7	23.5 §
						BF%	30.6%	30.5% §
					Usual care (*N* = 31)	Fat mass	26.9	27.8 *
						Trunk fat	15.2	15.5
						Visceral fat	926 g	922 g
						Lean mass	58.7	57.3 *
						ASM	24.9	24.3 *
						BF%	30.3%	31.4% *
Winters-Stone et al. [42]	RCT	Body composition	12 months 2 ×/week 165 min/week Supervised resistance at 60–80% 1 RM Impact exercise 1 ×/week Home-based exercise of choice	DXA	Exercise (*N* = 29)	Fat mass	24.3	23.9 §
						Trunk fat	13.5	13.1
						Lean mass	59.2	59.2
						BF%	28.7%	28.4%
					Flexibility (*N* = 22)	Fat mass	28.4	29.9
						Trunk fat	15.0	15.4
						Lean mass	57.5	57.2
						BF%	31.6%	32.4%
Wall et al. [43]	RCT	Cardiorespiratory fitness	6 months 2 ×/week 150 min/week Supervised aerobic at 70–90% HRmax Resistance exercise 6–12 RM, 1–4 sets 1 ×/week Home-based aerobic exercise	DXA	Exercise (*N* = 50)	Fat mass	24.1	24.5 §
						Trunk fat	13.2	13.0 §
						Lean mass	59.4	60.1 §
						BF%	27.2%	27.2% §
					Usual care (*N* = 47)	Fat mass	25.7	27.2
						Trunk fat	14.2	14.9
						Lean mass	58.7	58.6
						BF%	28.2%	30.3%
Newton et al. [36]	Details in aerobic section							
Ndjavera et al. [44]	RCT	Fat mass	12 weeks 2 ×/week Supervised aerobic at 55–85% HRmax Resistance exercise 10 RM, 2–4 sets Home-based aerobic exercise	BIA	Exercise (*N* = 24)	Fat mass	24.3	21.7
						Fat-free mass	58.2	58.9
					Usual care (*N* = 26)	Fat mass	23.3	22.7
						Fat-free mass	59.1	58.2

* = Significant within group change; § = significant between-group change; §UC = significant between-group change with usual care control group; §R = significant between-group change with resistance training group; # = effect of time in the two groups pooled together; §6DEL = significantly different to delayed/aerobic group at 6 months only, not 12 months which is the value reported in the table; *3 = significant loss at 3 months only, but not 6 months which is the value reported in the table. ^a^ Only reported mean change; ^b^ Acute ADT < 6 months, chronic ADT ≥ 6 months. RCT = randomised controlled trial; ×/week = times per week; HRmax = maximum heart rate; RM = repetition maximum; DXA = dual x-ray absorptiometry; ADT = androgen deprivation therapy; BF% = body fat percent; VO2max = oxygen consumption; MRI = magnetic resonance imaging; RPE = rate of perceived exertion; CT = computed tomography; ASM = appendicular skeletal muscle; BIA = bioimpedance analysis.

**Table 3:** Studies incorporating a nutrition component and assessed fat and lean mass in prostate cancer patients receiving ADT.

Study	Study Design	Primary Outcome	Intervention	Body Composition Assessment	Groups (*N*)	Outcome Variable	Mean Pre-Intervention Values (kg)	Mean Post-Intervention Values (kg)
Healthy eating guidelines and/or energy deficit								
O’Neill et al. [54]	RCT	Fat mass	6 months ≥5 ×/week 150 min/week Home-based brisk walking UK healthy eating guidelines + energy deficit diet if overweight.	Skinfolds	Intervention (*N* = 47)	Fat mass	28.8	26.9 §
						Lean mass	58.3	59.8
						BF%	32.6%	30.8% §
					Control (*N* = 47)	Fat mass	29.5	30.1
						Lean mass	59.8	59.1
						BF%	32.4%	32.8%
Gilbert et al. [52]	RCT	Brachial artery flow mediated dilatation	12 weeks 180 min/week 2 ×/week (1–6 weeks) 1 ×/week (7–12 weeks) Supervised aerobic at 55–75% HRmax + resistance exercise at 60% 1 RM 1 ×/week (1–6 weeks) 2 ×/week (7–12 weeks) Home-based exercise of choice Fortnightly healthy eating seminars	BIA	Intervention (*N* = 25)	Fat mass	34.5	31.6
						Skeletal muscle mass	31.9	32.9 §
					Usual care (*N* = 25)	Fat mass	30.4	29.6
						Skeletal muscle mass	31.2	32.3
Focht et al. [53]	RCT	Mobility	12 weeks 150 min/week 2 ×/week (1–6 weeks) 1 ×/week (7–8 weeks) Supervised aerobic 3–4 RPE (1–10 scale) + resistance 8–12 RM, 3 sets 1 ×/week (7–8 weeks) 2 ×/week (9–12 weeks) Unsupervised aerobic + resistance Home-based exercise of choice Nutrition counselling sessions—8 as a group and 2 individual phone calls + energy deficit diet if overweight.	Bod Pod	Intervention (*N* = 16)	Fat mass ^b^	−1.8 §	
						Fat-free mass ^b^	−0.06	
						BF% ^b^	−1.05% §	
					Usual care (*N* = 16)	Fat mass ^b^	0.9	
						Fat-free mass ^b^	−0.5	
						BF% ^b^	0.82%	
Freedland et al. [55]	RCT	Insulin resistance	6 months ≥5 d/week 150 min/week Home-based walking Carbohydrate intake ≤ 20 g/day	DXA	Intervention (*N* = 11)	Fat mass	32.3	24.0 §
						Lean mass	61.0	58.9 §
						BF%	28.3%	26.6% §
					Control (*N* = 18)	Fat mass	25.3	28.3
						Lean mass	55.9	55.4
						BF%	30.5%	32.3%
Baguley et al. [56]	RCT	Cancer-related fatigue and quality of life	12 weeks Individualised consultation with dietician every 2 weeks Mediterranean-style diet	DXA	Intervention (*N* = 12)	Fat mass	29.5	27.8 *
						Lean mass	53.2	52.0
					Usual care (*N* = 11)	Fat mass	29.8	29.3
						Lean mass	53.4	53.4
Wilson et al. [57]	Prospective cohort	Fat mass	12 weeks 3 ×/week 300 min/week Supervised resistance exercise at 6–12 RM, 2–4 sets Daily home-based aerobic exercise, RPE 3–8 (1–10 scale) 3 nutrition counselling sessions Calorie deficit diet 40 g protein powder after each supervised exercise session	DXA	Intervention (*N* = 14)	Fat mass	39.8	37.0 *
						Trunk fat	20.1	18.3 *
						Visceral fat	954 g	866 g *
						Lean mass	55.9	55.9
						ASM	23.3	23.3
						BF%	40.0%	38.3% *
Protein intake								
Dawson et al. [58]	RCT	Lean mass	12 weeks 3 ×/week 150 min/week Supervised resistance exercise at 60–83% 1 RM 2 × 25 g protein powder per day	DXA	Exercise (*N* = 8) + Exercise/protein (*N* = 8)	Fat mass	30.3	31.2
						Lean mass	48.5	53.2 §
						Fat-free mass	54.6	56.4 §
						ASM	23.5	24.8 §
						BF%	36.8%	35.9% §
					Protein (*N* = 10) + Flexibility control (*N* = 11) ^a^	Fat mass	25.6	26.2
						Lean mass	51.5	48.6
						Fat-free mass	51.4	51.5
						ASM	21.5	21.6
						BF%	33.9%	34.5%

* = Significant within group change; § = significant between-group change. ^a^ Patients were randomised to 4 groups: exercise, protein and exercise, protein, usual care control; however, for the analysis the two exercising groups and two non-exercising groups were combined as protein had no effect; ^b^ only reported mean change. RCT = randomised controlled trial; ×/week = times per week; RM = repetition maximum; DXA = dual x-ray absorptiometry; RPE = rate of perceived exertion; BF% = body fat percent; HRmax = maximum heart rate; BIA = bioimpedance analysis; UK = United Kingdom.

**Table 4:** Potential questions for future research relating to the prescription of exercise and nutrition for prostate cancer patients receiving ADT aiming to lose fat mass and gain lean mass.

	Unanswered Questions for Prostate Cancer Patients on ADT Aiming to Induce Fat Loss and Muscle Gain.
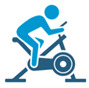 Aerobic training	1. Will a low-intensity lead-in period designed to build baseline fitness reduce injury risk and improve adherence, particularly for high-risk patients?
	2. Is there a minimum intensity/volume for lipolysis and muscle protein synthesis stimulation?
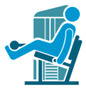 Resistance training	1. Will a low-intensity familiarisation period designed to build baseline strength reduce injury risk and improve adherence, particularly for high-risk patients?
	2. Is there a minimum intensity/volume for muscle protein synthesis stimulation?
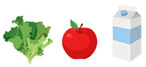 Nutritional intake	1. Who is an energy deficit or healthy eating guideline diet most appropriate for?
	2. What is the optimum protein intake to enhance muscle protein synthesis leading to muscle gain?
Other questions inclusive of all elements	1. Are the benefits gained from a combined exercise and nutrition intervention influenced by length of time on ADT?
	2. What is a clinically significant change in fat and lean mass for prostate cancer patients on ADT?

Images created with BioRender.com (accessed on 5 April 2021).

## Data Availability

Not applicable.
